# 

**Published:** 1915-09-04

**Authors:** 


### CONTENTS.

PAGE

**The Choice of Medicine as a Career** ... ... 461

**The Market Value of Hospital Service** ... 463

**Some Consequences of the War** ... ... ... 464

**Women in Pharmacy** ... ... ... ... ... 464

**Hospital and Institutional News** ... ... ... 465

The Need of more Women Doctors—Poor-Law Medical Officers and the Cost of Drugs—Sir William Thomas and Porth Cottage Hospital—Storing Coal for London Infirmaries—Cholera in Germany and Russia—The Yarmouth Hospital and the Guardians—Medical Students and the War—The "Collapse" of the Tuberculosis Service—Overcrowding, Poverty, and Small Ownership—The Advanced Insurance Consumptive and the Infirmary—Merthyr General Hospital—Canada's Splendid Co-operation: War Demands for the Wounded—Good Work in Norfolk—Medical Man as Day Labourer—This Week's Drug Market.

**Manchester's Misfortune** ... ... ... ... 469

The Retirement of Mr. Walter G. Carnt.

PAGE

**The Royal Army Medical Corps** ... ... ... 471

How Matters Stand To-day.

**The Royal Naval Medical Service** ... ... 474

In War and Peace.

**The Poor-Law Medical Service** ... ... ... 475

Its Hospital Branch.

**The Study of Tropical Medicine** ... ... 476

Some Colonial Office Requirements.

**Women's Future in Medicine** ... ... ... 477

Achievements and Opportunities.

**The Medical Curriculum** ... ... ... ... 479

Qualification and Registration.
The English Conjoint Course.
The Fellowship of the Royal College of Surgeons.
Oxford University Course.
Cambridge University Course.
The London University Course.
Higher Diplomas.

**The Medical Student and his Work** ... ... 482

The Medical Schools of the United Kingdom.

[*See over.*]

### CONTENTS—(*continued*).

| | PAGE |
|---|---|
| **The Public Services** ... ... ... ... ... ... | 488 |
| Royal Naval Medical Service. | |
| Royal Army Medical Corps. | |
| Indian Medical Service. | |
| Colonial Medical Service. | |
| **London Post-Graduate Institutions** ... .. | 489 |
| **Graduate Study in London Special Hospitals** | 4ΕΙ |
| **The Editor's Letter-Box** ... ... ... ... | xvi |
| "Infectious Disease" in Public Health (Scotland) Act, 1897. | |
| House Doctors and the War Office. | |
| Price of Coal (Limitation) Act, 1915. | |
| **Appointments to St. Thomas's Hospital** ... | xvi |
| **Scotch Charity Fetes in War-Time** ... ... | xviii |
| **The Late Dr. Archibald Douglas...** ... ... | xviii |
| **The Royal Sanitary Institute** ... ... ... | xviii |

